# **Construction of attapulgite decorated cetylpyridinium bromide/cellulose acetate composite beads for removal of Cr** (**VI**) **ions with emphasis on mechanistic insights**

**DOI:** 10.1038/s41598-024-62378-4

**Published:** 2024-05-28

**Authors:** Eman M. Abd El-Monaem, Ahmed M. Omer, Hesham A. Hamad, Abdelazeem S. Eltaweil

**Affiliations:** 1 Chemistry Department, Faculty of Science, Alexandria University, Alexandria, Egypt; 2 Polymeric Materials Research Department, Advanced Technology and New Materials Research Institute (ATNMRI), City of Scientific Research and Technological Applications (SRTA-City), P. O. Box: 21934, New Borg El-Arab City, Alexandria Egypt; 3 Fabrication Technology Research Department, Advanced Technology and New Materials Research Institute (ATNMRI), City of Scientific Research and Technological Applications (SRTA-City), P.O. Box 21934, New Borg El-Arab City, Alexandria Egypt; 4 Department of Engineering, College of Engineering and Technology, University of Technology and Applied Sciences, Ibra, 400, Sultanate of Oman

**Keywords:** Attapulgite, Cellulose acetate, Composite beads, Adsorption mechanism, Reusability, Environmental chemistry, Materials science, Nanoscience and technology

## Abstract

Eco-friendly and renewable composite beads were constructed for efficient adsorptive removal of Cr (VI) ions. Attapulgite (ATP) clay decorated with cetylpyridinium bromide (CPBr) was impregnated into cellulose acetate (CA) beads, which were formulated through a simple and cost-effective solvent-exchange approach. FTIR, XRD, SEM, Zeta potential, and XPS characterization tools verified the successful formation of ATP–CPBr@CA beads. The composite beads displayed a spherical and porous shape with a positively charged surface (26.6 mV) at pH 2. In addition, higher adsorption performance was accomplished by ATP–CPBr@CA composite beads with ease of separation compared to their components. Meanwhile, equilibrium isotherms pointed out that the Langmuir model was optimal for describing the adsorption process of Cr (VI) with a maximal adsorption capacity of 302 mg/g. Moreover, the D–R isotherm model verified the physical adsorption process, while adsorption data obeyed the pseudo-second-order kinetic model. Further, XPS results hypothesized that the removal mechanism involves adsorption via electrostatic interactions, redox reaction, and co-precipitation. Interestingly, the ATP–CPBr@CA composite beads reserved tolerable adsorption characteristics with a maximum removal present exceeding 70% after reuse for seven successive cycles, proposing its feasible applicability as a reusable and easy-separable candidate for removing heavy metals from aquatic bodies.

## Introduction

Chromium (Cr) are present in airborne dust, soil and water with high concentrations, causing prejudicial impacts on human health, marine life and the environment^1^. Chromium is categorized as one of the most notorious heavy metals that are considered the backbone of significant industries such as mining, fertilizer, smelting, pigments and electroplating^2^. Because of the persistent bioaccumulation and non-degradability of Cr ions, it can accumulate in human bodies, leading to teratogenicity, carcinogenicity and failure of many organs such as kidneys and liver. Noteworthy, Cr ions exist in two forms which are Cr (III) and Cr (VI)^3^. While Cr (VI) has more danger than Cr (III) since it has higher toxicity and a faster absorption rate by the human body^4^. Thereby, the world health organization has authorized that the permissible level of hexa-chromium in drinking water must not be more than 0.05 mg per litter^5^. Strikingly, the efficiency of water remediation strategies has been improved to counteract the risk of such detrimental ions. Among these strategies, adsorption is utilized on a large scale owing to its individual advantages in terms of efficiency, process rate, simplicity and energy-saving^6–8^.

Cellulose acetate (CA), the main member of the cellulose family, has recently drawn attention thanks to its applicability in bountiful fields including catalysis, batteries, food packaging, H_2_ and CO_2_ storage, drug delivery and wastewater treatment^9,10^. Besides, CA has suitable specifications for good adsorbents, but it suffers a serious bottleneck which is the low adsorption capacity^11^. For this sake, several studies have attested the incorporation of efficient substances into CA as a proper solution to this drawback^12,13^. The improvement of physicochemical characteristics and providing the unique functions can be achieved by various inorganic compounds^9^. Noticeably, polymer/clay composites are mouldable solid materials that are characterized by their excellent adsorption capacity, propitious thermal stability. In this context, many studies have recommended the incorporation of graphene oxide, clays such as hydroxyapatite, bentonite, zeolite and montmorillonite into CA as an auspicious solution to boost its properties and mainly the adsorption property^14–18^. However, there are a plethora of efficient clays that are not exploited to ameliorate the CA properties.

Among them, attapulgite (ATP) is one of the brilliant natural clays that has been widely applied in pivotal applications such as water remediation, agriculture, anticorrosion and catalysis^19^. ATP possesses remarkable advantages comprising cost-effective, biocompatibility, high specific surface and porosity, non-toxicity and high thermal stability^20^. In addition to the facile functionalization of ATP that eases its modification via active groups such as aminopropyl triethoxysilane, polyamine silane, poly dopamine, etc.^21–23^.

Cetylpyridinium bromide (CPBr) is a cationic surfactant that has exhibited excellent adsorption performance toward zwitterionic, anionic and cationic pollutants owing to its amphiphilic property^24,25^. Notably, the intercalation of CPBr into clay improves its core and surface since CPBr greatly increases the interlayer distance and also adsorbs onto the surface of clay, endowing it extra active sites^20,26,27^.

All of the above inspired us to fabricate the novel ATP–CPBr@CA composite beads that not only have an advanced adsorption property, but also an easy separation advantage. The constructed ATP–CPBr@CA beads and the pure substances were characterized via various tools. Further, the adsorption performance of ATP–CPBr@CA beads toward Cr (VI) was scrutinized in batch mode. Also, the selectivity of ATP–CPBr@CA bead towards Cr (VI) was evaluated in the existence of some common anions such as sulfate, nitrate and chloride. Moreover, it was a crucial issue to evince the recyclability of ATP–CPBr@CA bead via the reusability test for several repeated adsorption–desorption cycles. More importantly, the type of the Cr (VI) interactions with ATP–CPBr@CA beads were determined by kinetic and isotherm studies. Besides, the proposed mechanism of Cr (VI) adsorption onto the surface of ATP–CPBr@CA bead was also clarified.

## Experimental

### Materials

Cellulose acetate (molecular weight 30,000 g/mol, 39.8 wt% acetyl) and cetylpyridinium bromide (CPBr, 99%) were provided by Spectrum Chemical Co. (USA). Attapulgite (ATP, 99%), dimethyl sulfoxide (DMSO, 99%) and potassium dichromate (K_2_Cr_2_O_7_, 99%) were purchased from were bought from Alpha Chemika (India). Sodium chloride (NaCl, > 98%) and ethanol (C_2_H_5_OH, 99%) were provided by El Nasr Pharmaceutical Chemicals Company (Egypt).

### Fabrication of ATP–CPBr composite

ATP–CPBr composite was fabricated by ion-exchange approach^23^. A mixture of equal mass ratio from CPBr and ATP were dispersed into 50 mL distilled H_2_O and kept under stirring for three days at 35 °C. The resultant ATP–CPBr composite was collected, repeatedly eroded using dist. H_2_O and followed by drying for 10 h at 65 °C.

### Fabrication of ATP–CPBr@CA composite beads

**Scheme 1:**
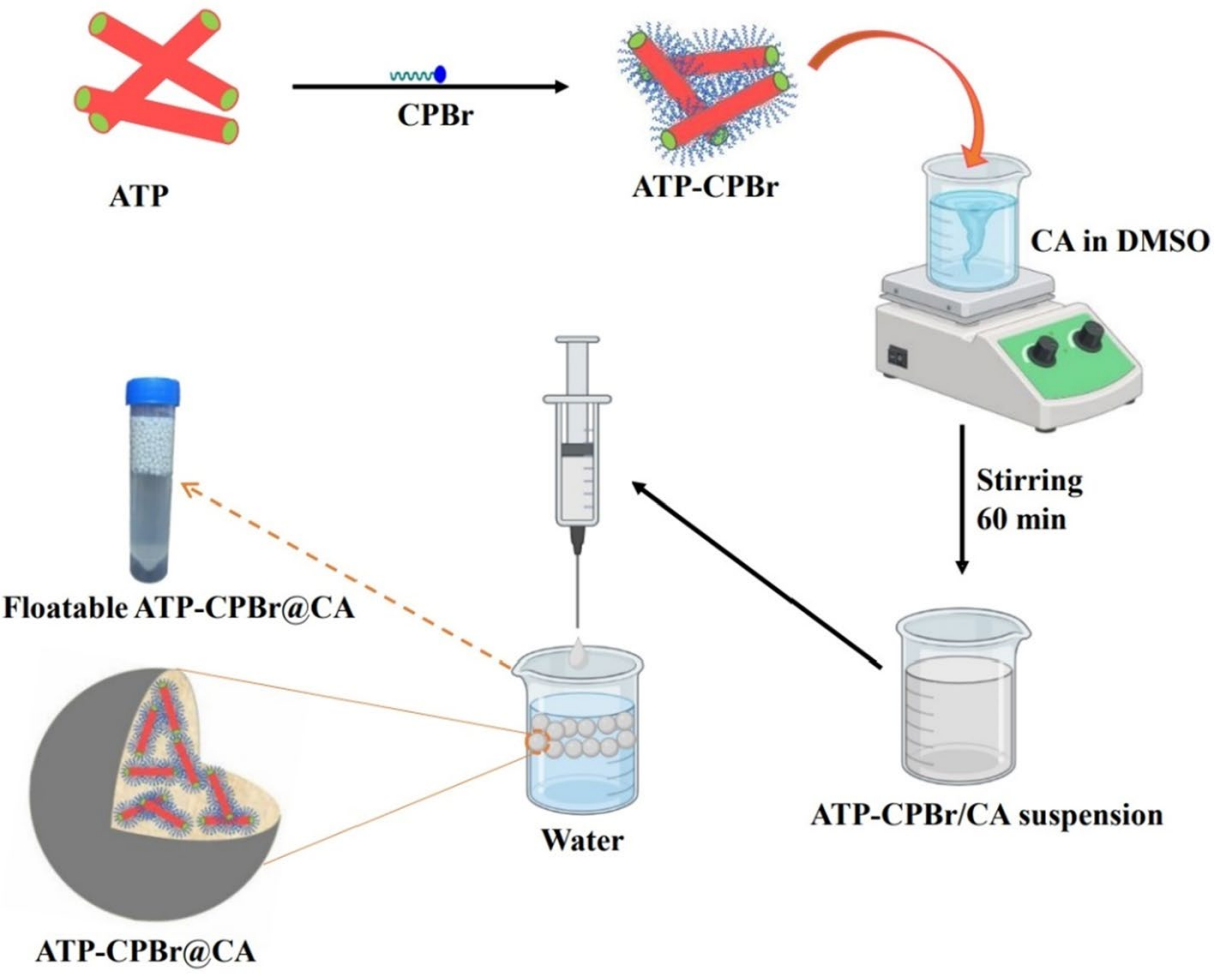
Representation of the ATP–CPBr@CA composite beads fabrication.

The as-fabricated ATP–CPBr was incorporated into CA beads as follows; dissolving 1 g of CA into DMSO under potent stirring for 60 min. Then, the CA solution added to 1 g of ATP–CPBr composite and agitated for 60 min. The obtained ATP–CPBr@CA composite was added to the distilled water (coagulant medium) by syringe under mild stirring. Finally, after the curing of ATP–CPBr@CA composite beads for 30 min, the beads were separated and washed by distilled H_2_O. Besides, CA beads were prepared via the same reported procedure in “Fabrication of ATP–CPBr@CA composite beads” section except for the step involving the addition of ATP–CPBr composite. Scheme 1 describes the preparation process of the adsorbent composite beads.

### Instrumental characterization

The details of characterization techniques are presented in Supplementary Information (S1).

### Batch adsorption studies

A series of batch experiments were performed to evaluate the adsorption process of Cr (VI) ions. To pick out the optimal pH, the removal of Cr (VI) by ATP–CPBr@CA adsorbent composite was examined at the pH range of 2–10. The influence of the ATP–CPBr@CA dose was examined at the dose range of 0.01–0.03 g. Moreover, the adsorption medium temperature was studied between 25 and 55 °C. The influence of the initial concentration of Cr (VI) was scrutinized at the concentration range of 50–300 mg/L. Besides, the selectivity of ATP–CPBr@CA towards Cr (VI) was evaluated in the existence of NO_3_^−^, Cl^−^ and SO_4_^2−^anions. After intervals of time (up to 180 min), the remaining Cr (VI) concentration was assayed at wavelength of 540 nm using spectrophotometric instrument. The removal (%) and adsorption capacity (q) were calculated as follow^28^:

1 $$Removal \% = \frac{{C_{0 } - C_{t} }}{{C_{0} }} \times 100$$

2 $$q = \frac{{(C_{0} - C_{t} ) \times V}}{m}$$

where *C*_*o*_ and *C*_*t*_ represent the concentration of Cr (VI) ions at initial and at time “*t*”, respectively. *V* and *m* are volume of Cr (VI) solution and mass of ATP–CPBr@CA beads, respectively.

### Recyclability test

To evaluate the recyclability of the developed composite beads, the beads were subjected for several adsorption–desorption runs. After each adsorption run, samples were collected from the Cr (VI) solution medium and subsequently soaked into a regenerative solution comprising NaCl/C_2_H_5_OH for 60 min. Finally, the regenerated samples were inspected for next adsorption run. The reusability experiment was conducted for seven successive cycles^29^.

## Results and discussion

### Studying the physiochemical properties of ATP–CPBr@CA

#### FTIR

**Figure 1:**
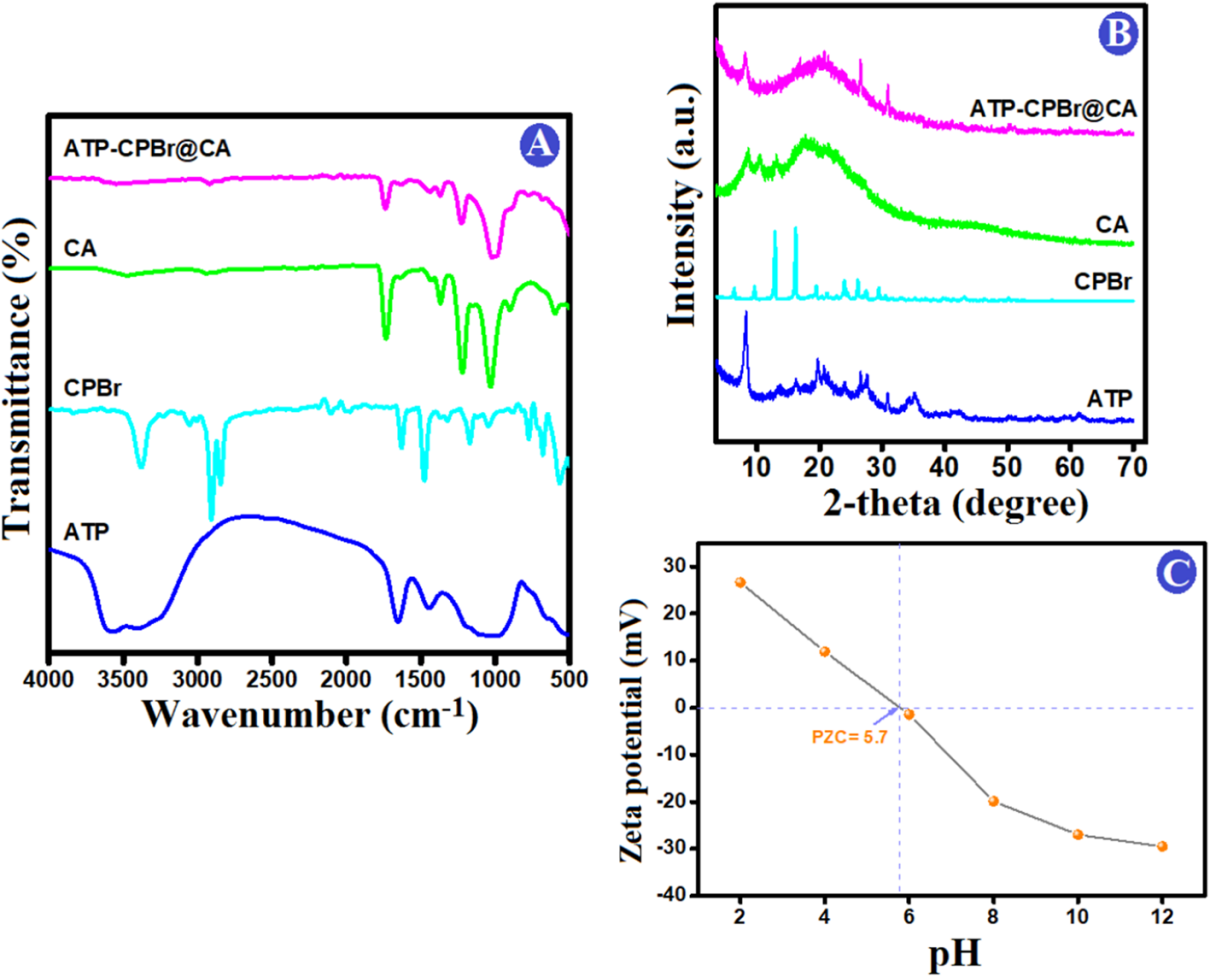
(**A**) FTIR spectra, (**B**) XRD patterns of ATP, CPBr, CA, and ATP–CPBr@CA, and (**C**) Zeta potential of ATP–CPBr@CA composite.

The utilization of FTIR spectra to determine the surface functional groups of the ATP, CPBr, CA and ATP–CPBr@CA composite before and after adsorption of Cr (VI) ions was revealed in Fig. 1A. As it can be observed that the main bands in the spectrum of ATP are located at 3610 cm^−1^ (for Si, Mg and Al stretching vibration), 3400 cm^−1^ (for OH stretching), 1661 cm^−1^ (–OH hydroxyl groups and adsorbed water), 1034 cm^−1^ (Si–O–Si stretching)^30,31^. FTIR spectra of CPBr observed the absorption bands at 3403, 2943, 1661, 1466, and 1003 cm^−1^ which could be assigned to the O–H stretching, asymmetric CH_2_ stretching, –OH bending, C–H bending, O–H of the distortion peak of H_2_O, and C–N stretching, respectively^32,33^. The spectrum of CA shows peaks at 1730, 1461, 1351, 1237, 1168 and 1007 cm^−1^ which corresponded to C=O stretching, C–CH_3_ symmetric and asymmetric deformation, C–C–O stretching, C–O–C in cellulose chain, C–O stretching bridge, respectively^26,34^. A comparison between the original and ATP–CPBr@CA composite, FTIR spectra demonstrated that the latter featured peaks is obtained in the composite but there were significant weakened in the intensity which potentially confirmation the reaction between ATP and CA and CPBr and proving the successful fabrication of hybrid composite via electrostatic interaction at the surface (Vander Walls forces). Also, the positions of the peaks is influenced by the change of inter and intermolecular hydrogen bonding, and therefore related to the changes in the chemical surface groups. In summary, FTIR studies indicated that the ATP–CPBr@CA composite has various functional groups including –OH, C–O, C=O and Si–O that provided a significant role in boosting the adsorptive removal of Cr (VI) by the ATP–CPBr@CA composite beads. Also, there is shift in the position of peaks in ATP–CPBr@CA before and after rejection of Cr (VI) that would be clarified in the section of removal mechanism.

#### XRD

Figure 1B depicts the crystallographic patterns of ATP, CPBr, CA, and ATP–CPBr@CA beads. The ATP pattern illustrates its distinctive peaks at 2-theta 8.2°, 13.7°, 16.2°, 19.8°, 20.6°, 24.2°, 27.6°, and 35.1°, while the peaks at 2-theta 26.7° and 30.8° are ascribed to the presence of quartz and dolomite impurities, respectively^21^. The CPBr pattern showed the belonging peaks at 2-theta 6.3°, 9.6°, 12.8°, 16.1°, 19.1°, 24.1°, 26.1°, 27.2°, and 29.2°. The pattern of CA shows its amorphous character with a distinguishing broad XRD peak at 2-theta 20°^35^. The XRD crystallographic pattern of ATP–CPBr@CA reveals the amorphous phase of the composite beads with the appearance of some characteristic peaks to CTP and CPBr. This observation implied the core–shell structure of ATP–CPBr@CA in which the CA shell shielded the core diffraction.

#### Zeta potential

In order to detect the surface charges of ATP–CPBr@CA hybrid composite beads, the zeta potentials were evaluated. Figure 1C shows that the point of zero charge was 5.7, since the maximum of surface potential was 26.6 mV at pH = 2 and then reduced gradually to -29.28 mV at pH = 12. The surface of ATP–CPBr@CA composite becomes neutral at pH = 5.7. As a result, it displays favourable results in acidic solutions (pH 2–5). When pH increases from 6 to 12, the production of ATP–CPBr@CA species causes the surface charge to turn negative. As a result, zeta potential may have decreased when pH was more than 6 due to the deprotonation of ATP–CPBr@CA surface groups or the adsorption of OH on ATP–CPBr@CA surface. Lower pH values (pH 2–5) rendered the surface of ATP–CPBr@CA to become positively charged which attracted the anionic Cr_2_O_7_^2−^ and HCrO_4_^−^ species by a potent electrostatic interaction.

#### Morphological characteristics

**Figure 2:**
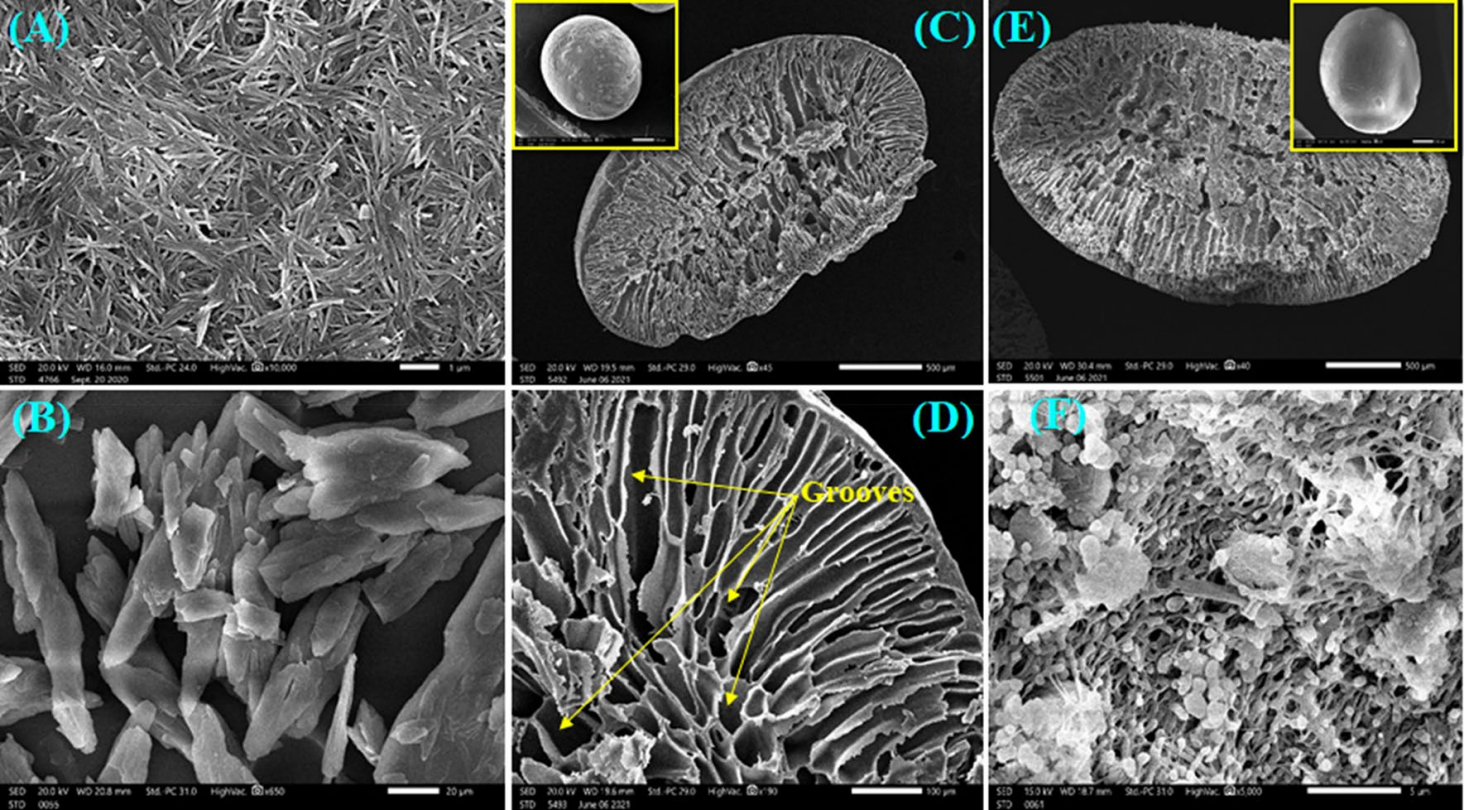
SEM of (**A**) ATP, (**B**) CPBr, (**C**) CA beads (low magnification), (**D**) CA beads (high magnification), (**E**) ATP–CPBr@CA beads (low magnification) and (**F**) ATP–CPBr@CA composite beads (high magnification).

The morphological characteristics of ATP, CPBr, CA, ATP–CPBr@CA composite are shown in Fig. 2. The prepared ATP has regular shape of individual rods or aggregated of many rods (Fig. 2A), while the SEM image of CPBr showed irregular shape of rough and dense surface (Fig. 2B). CA beads (Fig. 2C) represented as spherical, smooth, and hard beads with a well-defined shape. Although CA beads appeared to be solid without any micro pores on their surface, the cross-section investigation showed a notable porosity at the microspcopic scale in the grooves form (Fig. 2D). After incorporation of ATP–CPBr into CA beads (Fig. 2E), it also observed as a spherical and smooth surface. However, the cross-section of composite beads showed network of microfibers containing beads. The high magnifications revealed that the surface of CA was modified with ATP–CPBr (Fig. 2F). In summary, the SEM images verified that ATP–CPBr composite was successfully incorporated into CA beads.

#### Surface chemistry characteristics

**Figure 3:**
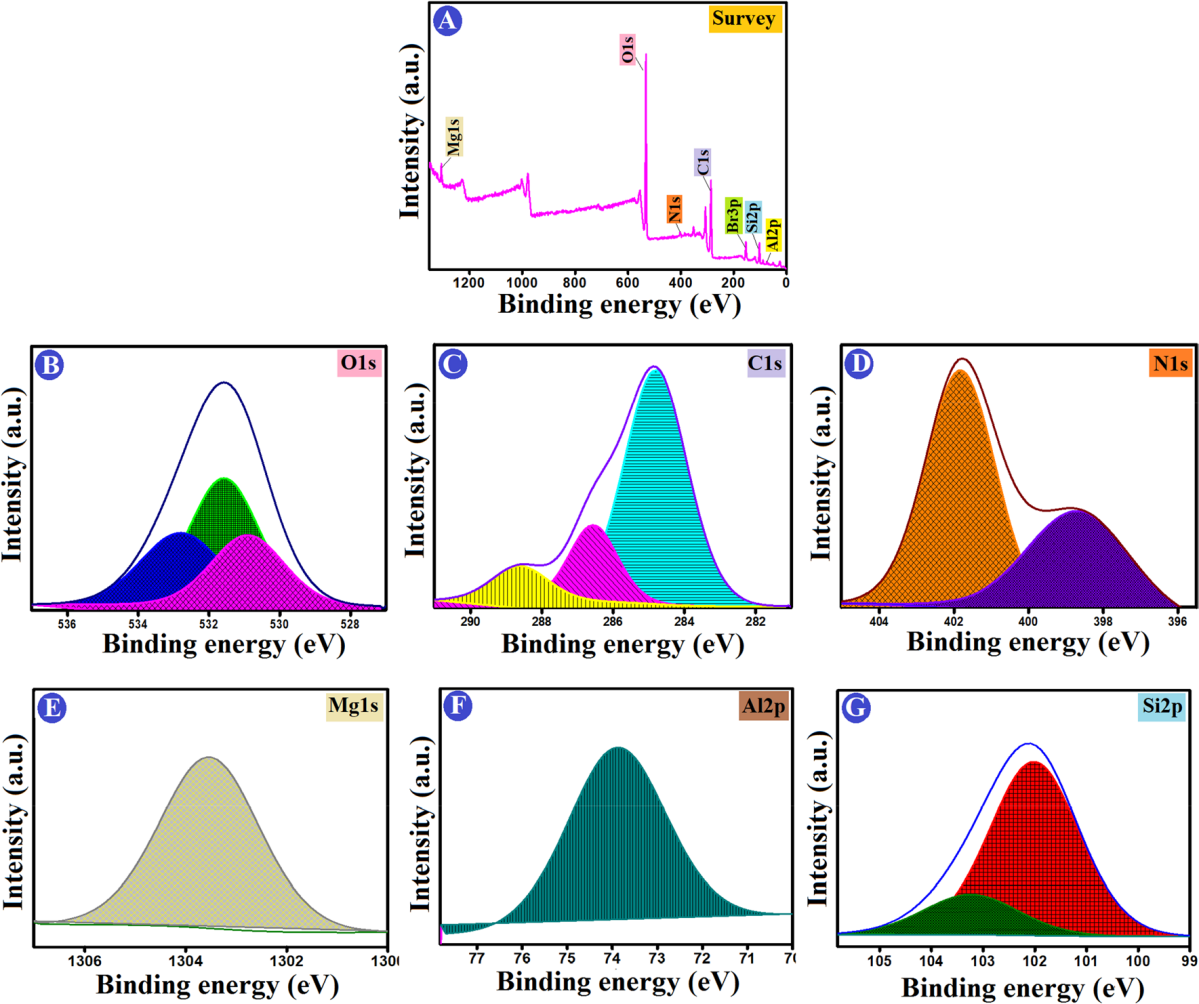
XPS spectra of (**A**) a wide scan, and deconvolution of (**B**) O_1*s*_, (**C**) C_1*s*_, (**D**) N_1*s*_, (**E**) Mg_1*s*_, (**F**) Al_2*p*_, and (**G**) Si_2*p*_ of ATP–CPBr@CA composite beads.

To gain insight into the formation mechanism of ATP–CPBr@CA composite, the interaction of surface groups was confirmed by XPS spectra. Low resolution spectra show the main characteristic of Al, Si, Mg, C, O, and N at the surface of ATP–CPBr@CA before and after adsorption (Fig. 3A). In details, the peak of O1s is deconvoluted into three signals with centres at 530.9, 531.58, and 532.8 eV. These signals can be attributed to surface lattice oxygen (O_2_), chemisorbed oxygen species in surface oxygen vacancies, also known as (O–, O_2_^−^, or O_2_^2−^), and oxygen-containing groups like H_2_O, –OH^−^, or –CO_3_^−^ (Fig. 3B)^36,37^. Related to C_1*s*_ deconvolution, it indicated that carbon atoms in different surface functional groups: *sp*^2^ carbon at 284.8 eV (non-oxygenated carbon ring), C–O and C–N at 286.56 eV, and O–C=O at 288.62 eV^38^ (Fig. 3C). The deconvolution of N_1*s*_ indicating two types of N species are pyridinic at 398.67 eV and graphitic N at 401.81 eV (Fig. 3D)^39^. The binding energy of Mg element was 1303.53 eV that matching with MgO (Fig. 3E), while the binding energy of Al element was 73.89 eV which could be attributed to the existence of Al_2_O_3_ (Fig. 3F)^40^. The typical emission peak of Si–O–Si was visible in the XPS spectra of the Si _2*p*_ region at 102.02 eV. The signal at 103.24 eV, however, matches well with siloxy species like SiO_2_ (Fig. 3G).

### Adsorption characteristics

#### Comparison study

**Figure 4:**
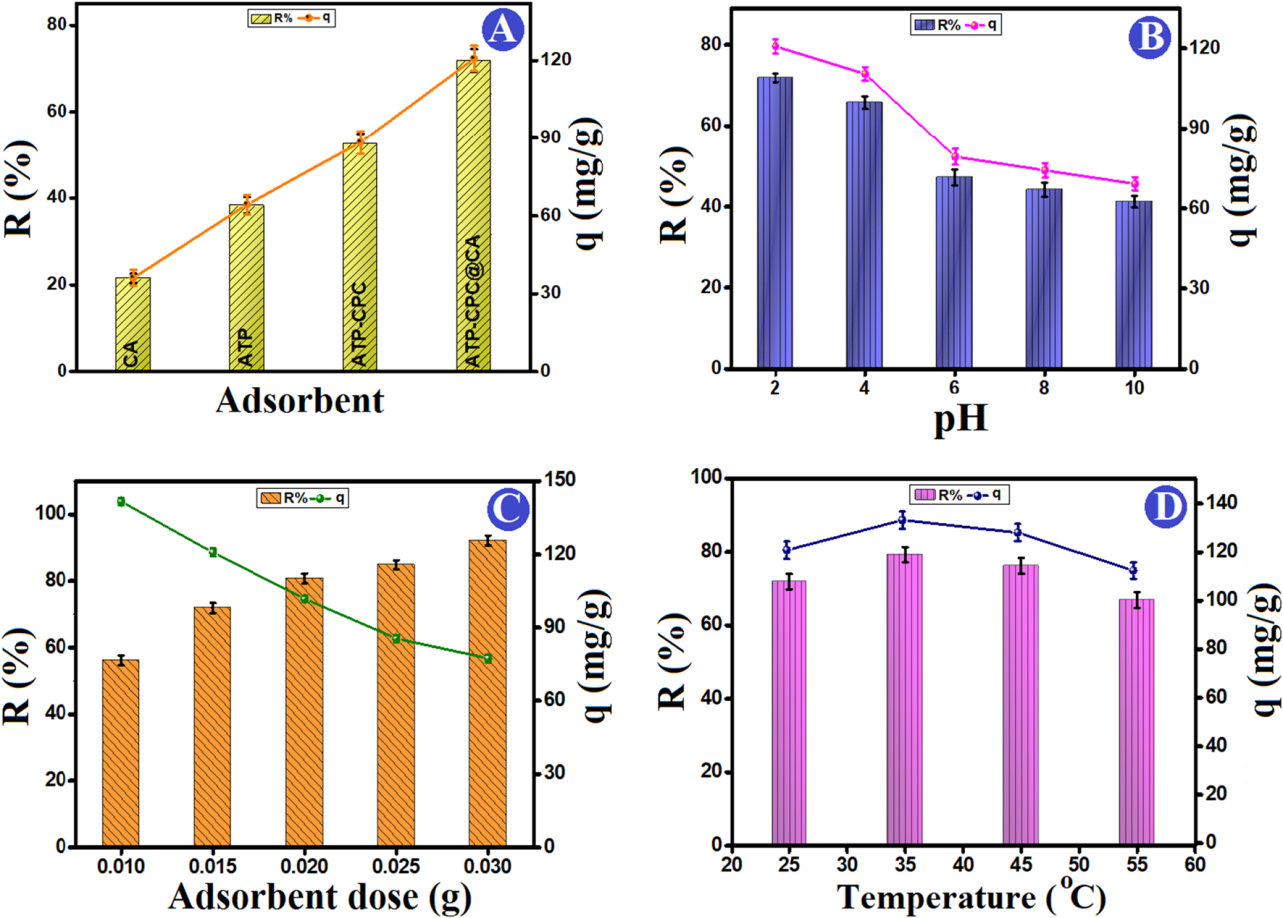
(**A**) Affinity of beads composition for removal of Cr (VI) ions, (**B**) impact of pH, (**C**) adsorbent dosage, and (**D**) system temperature on the removal (%) and the adsorption capacity of Cr (VI) onto ATP–CPBr@CA composite beads at constant [contact time 60 min, initial Cr (VI) concentration 100 mg/L, and temperature 25 °C].

The removal efficiency of ATP, CA, ATP–CPBr and ATP–CPBr@CA toward the adsorptive removal of Cr (VI) was investigated, as represented in Fig. 4A. It was recorded that the removal % of Cr (VI) into CA, ATP, ATP–CPBr, and ATP–CPBr@CA were 21.46%, 38.25%, 52.46%, and 71.88%; in addition, their adsorption efficacies toward Cr (VI) were 36.09 mg/g, 64.29 mg/g, 88.35 mg/g, and 120.81 mg/g, respectively. These findings denoted the synergistic effect between the authentic components to form higher efficacious ATP–CPBr@CA composite beads.

#### Effect of pH

Figure 4B displays the findings of an investigation into how the pH of the original solution affected the effectiveness of removing Cr (VI) from an aqueous solution using composite beads made of ATP–CPBr@CA. It can be observed that pH levels greatly influence the Cr (VI) adsorption onto ATP–CPBr@CA composite beads. With an increase in pH from 2 to 12, the adsorption of Cr (VI) ions onto the surface of ATP–CPBr@CA composite beads significantly reduced from 100 to 60 mg/g, in accordance with the literature^41^. At highly acidic conditions (pH = 2), the removal efficiency of Cr (VI) is found to be significantly reduced as pH increases. In an aqueous solution lower than pH = 4, it may to form the various ionic forms of Cr (VI) such as HCrO_4_^−^, CrO_4_^2−^, Cr_2_O_7_^2−^, Cr_3_O_10_^2−^, and Cr_4_O_13_^2−^ with predominance of HCrO_4_^−^. On the other hands, by increasing the pH above 4, it was shifted to formation of CrO_4_^2−^ which supports the electrostatic interaction between CrO_4_^2−^ and NH_3_^+^on the surface of ATP–CPBr@CA^42^. According to the literature, HCrO_4_^−^ is more easily adsorbed than CrO_4_^2−^ as a result of the low adsorption surface free energy^43^. Moreover, at lower pH solutions, the exchange of Br^−^ anion of CPBr with anionic Cr (VI) in the adsorption medium was the main source of Cr (VI). Also, it was hypothesized that the Cr (III) was formed when Cr (VI) was reduced by electrons from ATP–CPBr@CA, resulting in the lower pH that was observed^44^.

On the other hands, at pH higher than 6, there are more negative charges on the surface of ATP–CPBr@CA that support the electrostatic repulsion between ATP–CPBr@CA and Cr (VI), making the adsorption of Cr (VI) onto ATP–CPBr@CA surface is difficult. The solution becomes alkaline when CrO_4_^2−^ was the dominating species, and OH^−^ then competed with Cr (VI) on ATP–CPBr@CA. Moreover, two exchange sites were filled by the CrO_4_^2−^ species, which exchanged with two Cl^−^ molecules, reducing the maximum amount of Cr ions that could be absorbed by ATP–CPBr@CA. All of these factors resulted in reduced Cr (V) adsorption at higher pH levels. As a result, the surface chemistry at the interface may be used to describe the influence of solution pH on the adsorption process^45^. At normal pH range, all forms of Cr (VI) are negatively charged, regardless of how dominant they are. The functional groups on ATP–CPBr@CA have a lone-pair of electrons from N atom, which mainly contribute as an active site for the formulation of ATP–CPBr@CA–Cr complex.

Further specific examples of how pH affects the adsorption process can be provided using the zeta potential. The surface of the adsorbent is positively charged when the pH of the solution is below 6, and negatively charged when the pH is above 6. As a result, it’s possible that the positive surface of ATP–CPBr@CA composite beads in an acidic media is more appealing to Cr (VI). By other means, as the pH of the adsorption medium declined, the density of positively charged sites increased, possibly as a result of electrostatic interactions between the anionic Cr (VI) species and the positively charged ATP–CPBr@CA composite. However, as the pH of the solution increased, the anionic binding sites on the ATP–CPBr@CA surface led to an increment in the electrostatic repulsion between the anionic Cr (VI) species and the beads, resulting in a substantial reduction in the adsorption amount of Cr (VI).

#### Impact of dose

Figure 4C illustrates the adsorption of Cr (VI) as a function of the dose of ATP–CPBr@CA at constant concentration, pH, and temperature. The acquired data depict that the removal efficiency of Cr (VI) ions was significantly boosted with increasing the dosage of ATP–CPBr@CA owing to the ample available binding sites to adsorb Cr (VI) species. On the contrary, the adsorption capacity decreased from 141.71 to 77.43 mg/g, as the ATP–CPBr@CA dosage increased from 0.01 to 0.03 g because of the augmentation of the un-occupied active adsorption sites with elevating the ATP–CPBr@CA proportion at a constant Cr (VI) concentration^46^.

#### Impact of solution temperature

Figure 4D shows how different temperatures affected the effectiveness of removing Cr (VI) ions. With increasing the adsorption medium temperature 25–35 °C, the removal efficiency of Cr (VI) improved, showing that the high temperature facilitated the adsorption process and subsequently accelerated the adsorption capability of Cr (VI)^47^. The increased rate of Cr (VI) removal can be explained by: (1) the enhanced ATP–CPBr@CA mass transfer caused by the Cr (VI) ions’ increased reactivity and diffusion rate, and (2) the thinner boundary layer surrounding the ATP–CPBr@CA, which helps to accelerate the adsorption process^48^. Temporarily, as the reaction between ATP–CPBr@CA and Cr (VI) is an endothermic chemical process, a higher temperature was also advantageous for the elimination of Cr (VI) ions^49^. Above 35 °C, there is a reduction in the adsorption of Cr (VI) which is related to the desorption originated from boosting the thermal energy of Cr (VI) ions^50^.

### Adsorption mechanism of Cr (VI)

#### Kinetics studies

**Figure 5:**
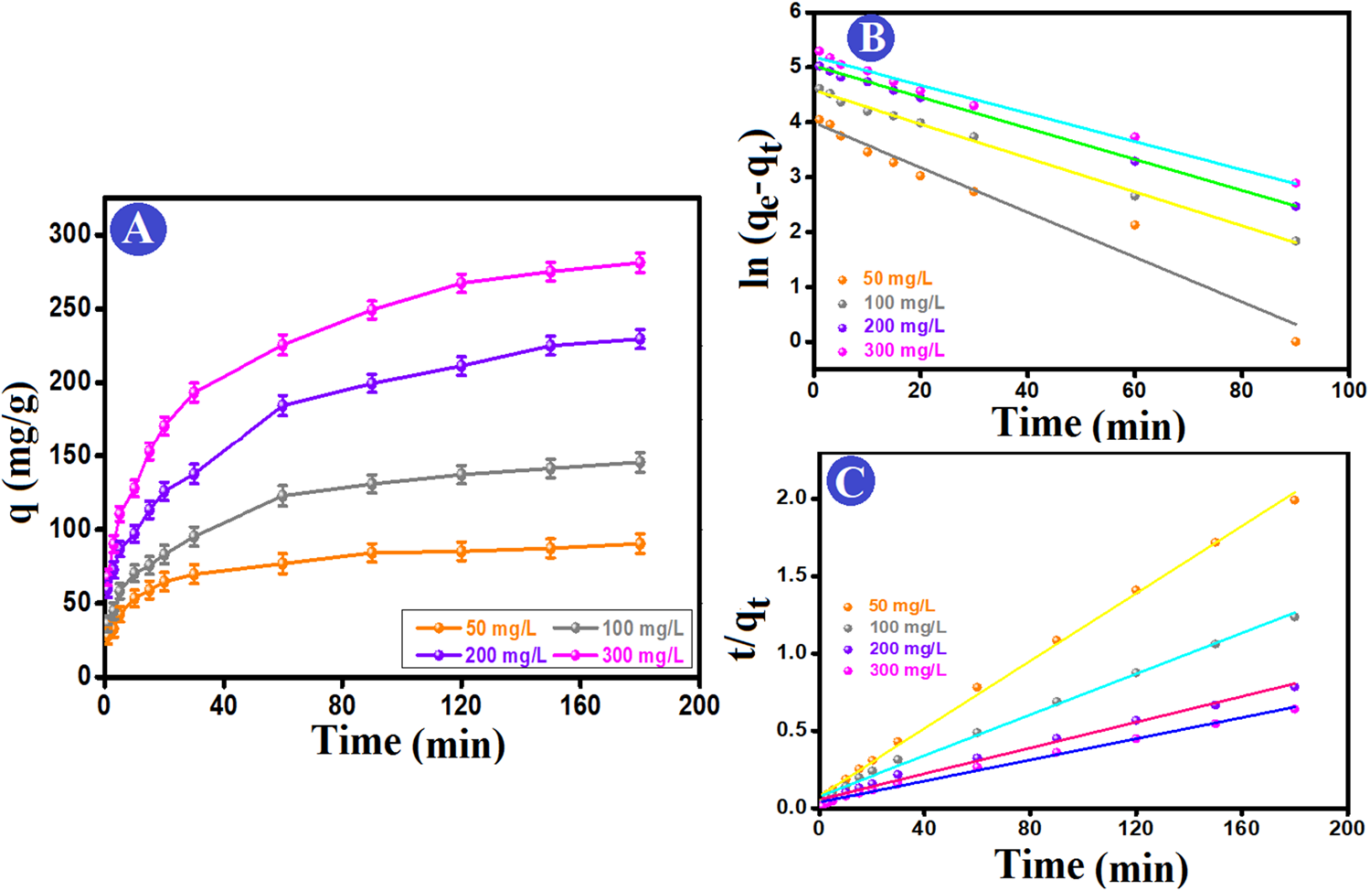
(**A**) Impact of initial concentration on the adsorption capacity of Cr (VI) onto ATP–CPBr@CA composite beads, (**B**) pseudo 1st order kinetic model, and (**C**) pseudo 2nd order kinetic model.

The impact of the initial Cr (VI) concentrations was investigated in the range of 50–300 mg/L during 180 min (Fig. 5A). The highest adsorption capacity of Cr (VI) ions was 281.20 mg/g at an initial Cr (VI) concentration of 300 mg/L, using 0.015 g of the ATP–CPBr@CA beads. The findings revealed that under high concentrations of Cr (VI) ions, the active sites of ATP–CPBr@CA would be effectively used. Further, the adsorption rate rises initially before gradually decreasing the duration of the adsorption time till reaches the equilibrium state after 120 min. It is difficult to occupy any remaining unoccupied adsorption sites due to the repulsive interaction between adsorbed Cr (VI) ions and those exist in the bulk phase after the equilibrium period^51^.

**Figure 6:**
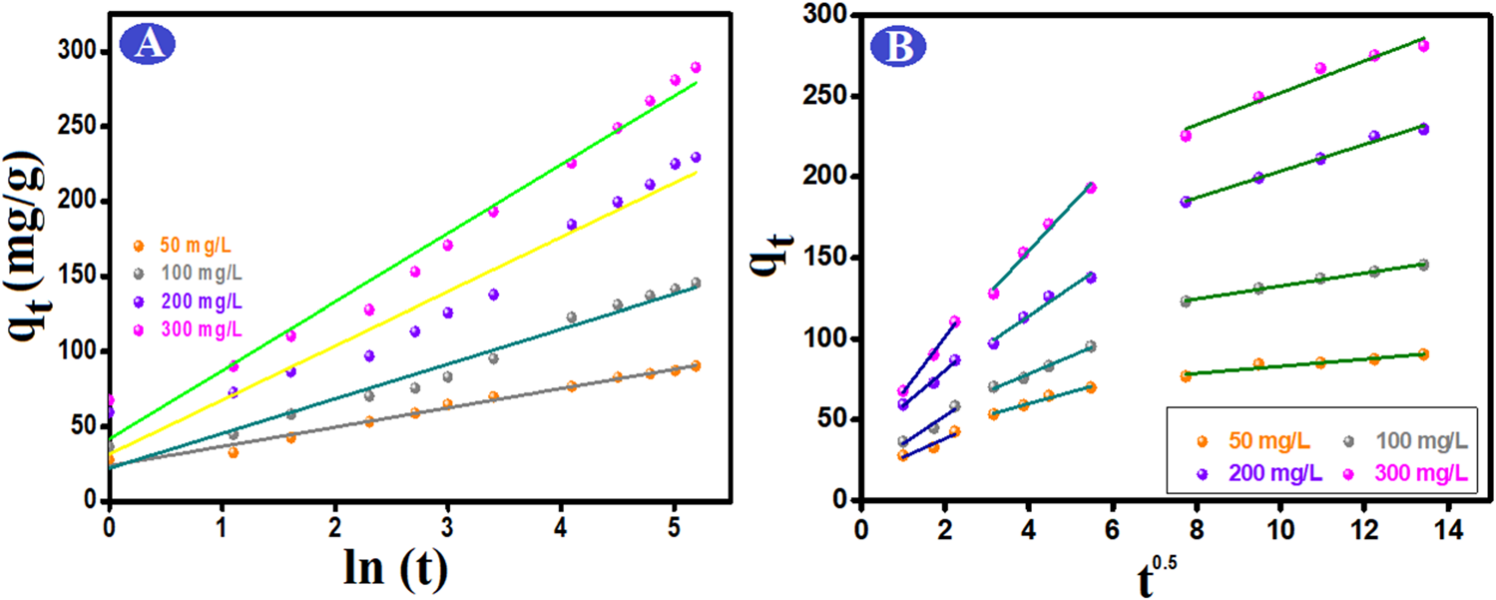
(**A**) Elovich and (**B**) intraparticle diffusion kinetic models of the Cr (VI) removal by ATP–CPBr@CA composite beads.

To understand and predict how reaction time would affect the retention and mobility of Cr (VI), the process was scrutinized using the adsorption kinetics. The investigation into the kinetics of Cr (VI) adsorption onto ATP–CPBr@CA at various initial concentrations provided the results shown in Fig. 5B,C for Pseudo first and Pseudo second order, respectively, and Table 1. The adsorption process attained the equilibrium within 120 min. The correlation coefficients of the Pseudo 2nd order model (R^2^ = 0.998) are greater than those of the Pseudo 1st order model (R^2^ = 0.953). Furthermore, there are an analogy between the computed adsorption capacities by Pseudo 2nd order at varies concentrations of Cr (VI) and the actual adsorption capacities. Such observations denoted the appropriateness of Pseudo 2nd order to model the Cr (VI) adsorption onto ATP–CPBr@CA and the domination of the chemical interactions in the adsorption process^52^. Interestingly, Elovich (Fig. 6A) implied the greater rate of adsorption compared to the rate of desorption, where the values of α are larger than β values at the varied concentrations of Cr (VI)^53^.

**Table 1:** Estimated constants of the kinetic parameters of Cr (VI) on ATP–CPBr@CA composite beads.

Kinetic models and parameters	Concentration (mg/L)			
	50	100	200	300
q_e, exp_(mg/g)	85.17	137.09	211.09	267.29
Pseudo 1st order				
q_e,cal_ (mg/g)	54.19	98.07	152.49	179.59
k_1_ (min^−1^)	0.040	0.030	0.028	0.025
R^2^	0.953	0.995	0.995	0.986
Pseudo 2nd order				
q_e,cal_ (mg/g)	91.74	151.52	240.38	294.11
k_2_ (g mg^−1^ min^−1^)	0.0015	0.0005	0.0003	0.0002
R^2^	0.998	0.995	0.991	0.994
Elovich				
α (mg/g min)	82.43	60.45	86.37	113.66
β (g/mg)	0.078	0.042	0.028	0.022
R^2^	0.987	0.966	0.942	0.973

Moreover, the intraparticle diffusion kinetic model was applied to predict the diffusion pathway of Cr (VI) from the bulk solution to the ATP–CPBr@CA surface. Figure 6B demonstrated that the Cr (VI) migration pathway to the adsorption groups of ATP–CPBr@CA took place throughout three stages; in the first step, Cr (VI) emigrated gradually from their solution and occupied the active sites on the surface of ATP–CPBr@CA. In the second stage, the ions began to get through the pores of ATP–CPBr@CA. Ultimately, in the last step; the Cr (VI) ions permeated the interior pores of ATP–CPBr@CA until attained equilibrium. From Table 2, increasing the concentration of Cr (VI) ions leads to increasing the driving forces that facilitate the intraparticle diffusion of Cr (VI) ions onto ATP–CPBr@CA composite beads. Also, compared to the first zone (related to film diffusion, C1), the second region (related to intraparticle diffusion, C2) had a thicker boundary layer. The plot does not pass through the origin confirming the intra-particle diffusion is not only the rate-controlling step^54^.

**Table 2:** The kinetic parameters from intraparticle diffusion model.

C_o_ (mg/L)	First step			Second step			Third step		
	K_p_,1	C_1_	R^2^	K_p_,2	C_2_	R^2^	K_p_,3	C_3_	R^2^
50	11.58	15.15	0.826	7.16	31.23	0.973	2.18	61.22	0.905
100	17.20	17.95	0.895	10.96	34.53	0.984	3.98	92.59	0.993
200	21.81	36.81	0.970	17.50	44.11	0.957	8.25	121.18	0.986
300	34.39	32.05	0.988	28.05	42.05	0.980	9.88	153.22	0.948

#### Isotherm study

**Figure 7:**
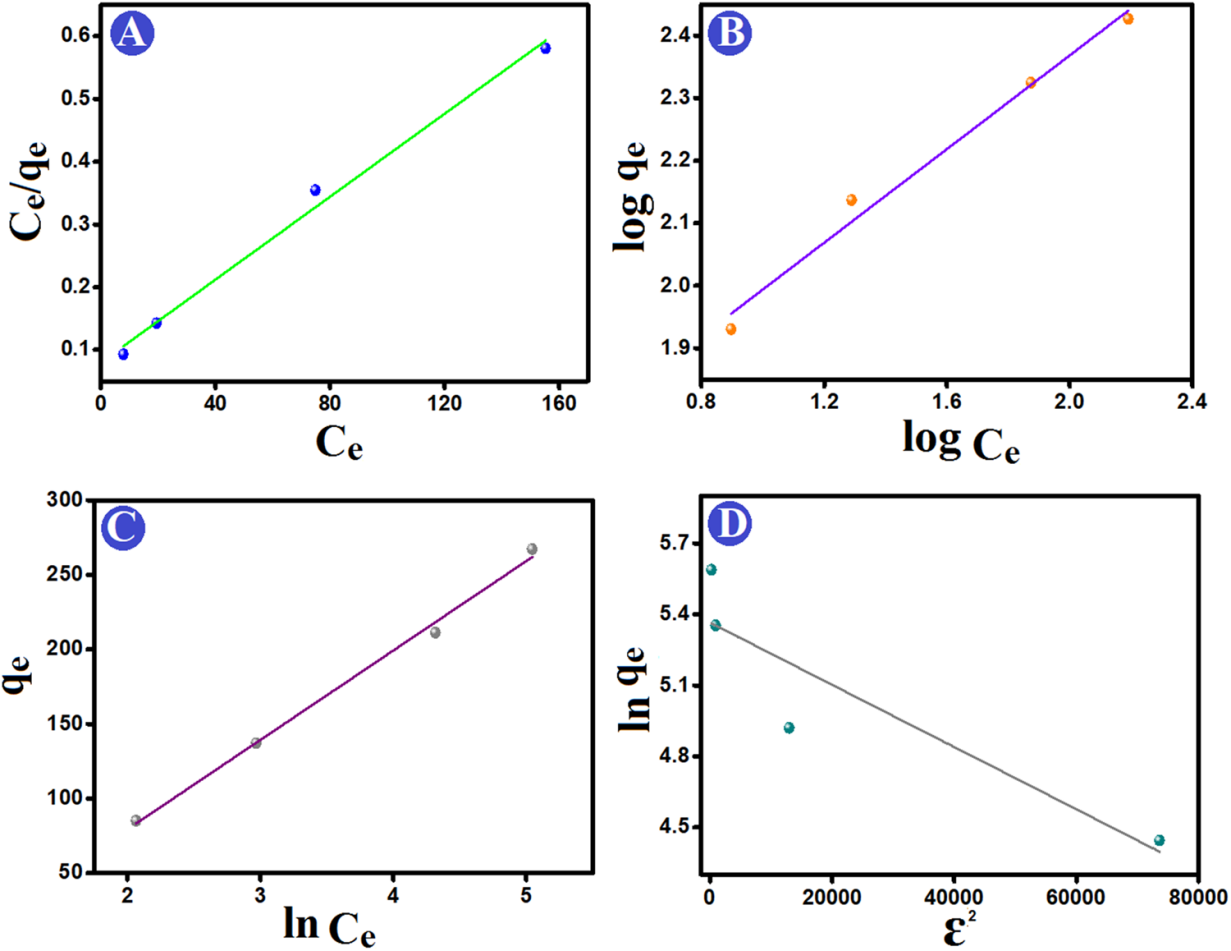
Equilibrium isotherm models of Cr (VI) adsorption onto ATP–CPBr@CA; (**A**) Langmuir, (**B**) Freundlich, (**C**) Temkin, and (**D**) D–R adsorption isotherm models.

For inferring the interactions’ nature between Cr (VI) and ATP–CPBr@CA, the resultant equilibrium data were modelled by Langmuir, Freundlich, Temkin, and D–R (Fig. 7A-D). Generally, the Langmuir model supposes the adsorption of the contaminant species onto the adsorbent surface via the formation of chemical interactions between them, producing a monolayer of the contaminants over the surface of the adsorbent^55^. Moreover, the Freundlich model postulates proceeding the contaminants adsorption via the occurrence of multi-layer physical interactions between the contaminant species and the adsorbent^56,57^. On the other hand, the Temkin and the D–R models could identify if the controlling interactions between the contaminant species and the adsorbent are physical or chemical based on b and E values, respectively.

In light of the acquired isotherm parameters (Table 3), the Cr (VI) uptake process was well-represented via Langmuir (R^2^ = 0.989) and Freundlich (R^2^ = 0.985) models. This result denoted the implication of both physical and chemical interactions in the uptake of Cr (VI) onto ATP–CPBr@CA. Moreover, the maximal Cr (VI) uptake capacity by Langmuir was 302.11 mg/g. The n value of Freundlich reflected the surface suitability of ATP–CPBr@CA to adsorb Cr (VI) species. Notably, the derived b value from Temkin supposed the physisorption of Cr (VI) onto ATP–CPBr@CA since b was lower than 80 kJ/mol. This observation agreed with D–R which also implied the controlling physical interaction on the Cr (VI) adsorption, where E was lower than 8 kJ/mol.

**Table 3:** Adsorption equilibrium isotherm parameters for Cr (VI) adsorption onto ATP–CPBr@CA composite beads.

Isotherm model	Parameter	Value
Langmuir	q_m_ (mg/g)	302.11
	b (L/mg)	0.041
	R^2^	0.989
Freundlich	n	2.66
	k_F_ (L/mg)	41.59
	R^2^	0.978
Temkin	A (L/g)	0.50
	B (J/mol)	59.99
	b (KJ/mol)	0.041
	R^2^	0.993
D–R	q_s_	213.81
	K_ad_ (mol^2^/K^2^J^2^)	1.31 × 10^–5^
	R^2^	0.747
	E (kJ/mol)	0.195

#### XPS analysis

**Figure 8:**
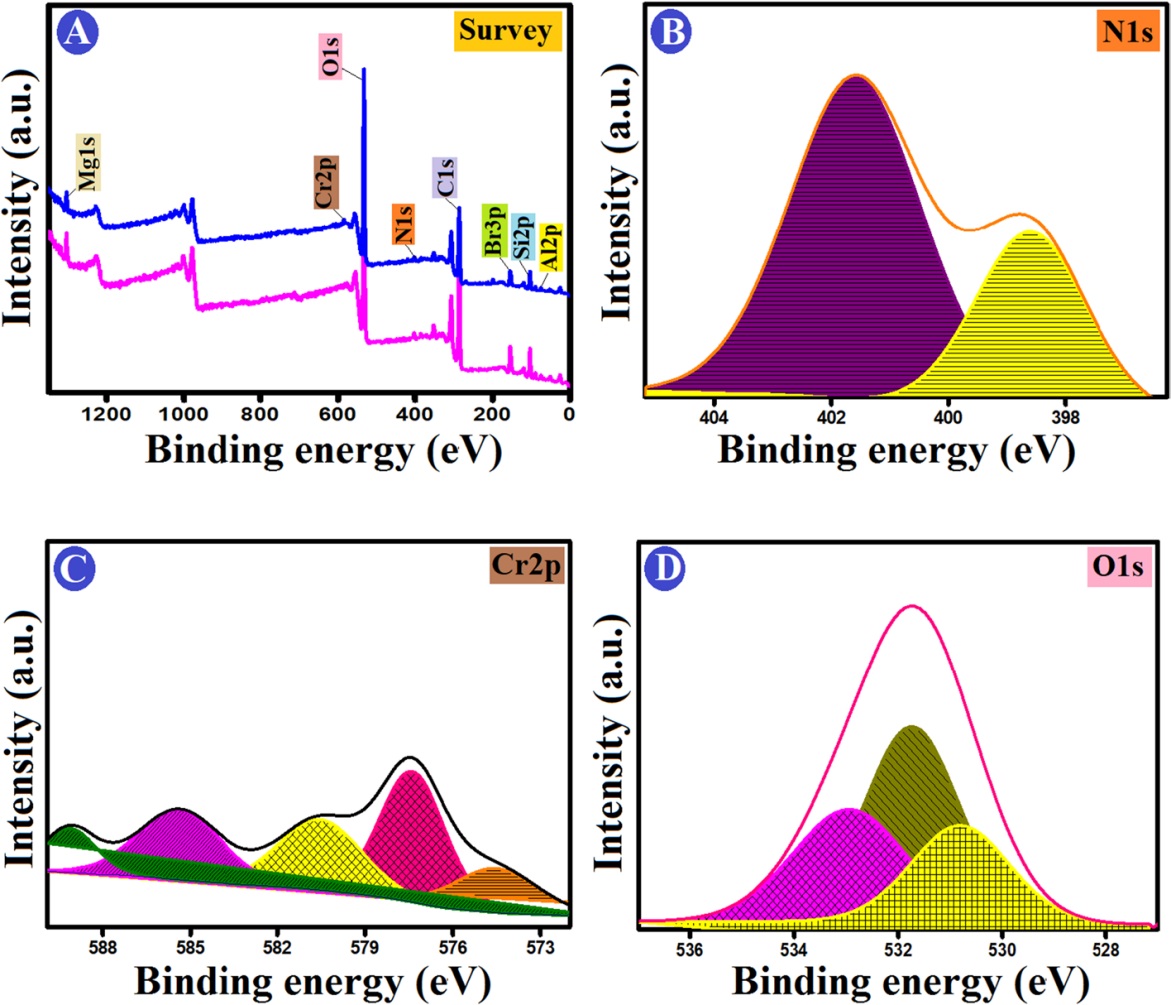
XPS of Cr (VI)-loaded ATP–CPBr@CA; (**A**) Survey, (**B**) N1*s*, (**C**) Cr2*p*, and (**D**) O1s.

According to kinetics and isotherms, the Cr (VI) adsorption onto ATP–CPBr@CA occurred via physical and chemical interactions. Hence, XPS spectra were used to predict how these interactions proceeded in detail. The XPS survey of Cr (VI)-loaded ATP–CPBr@CA revealed the distinctive peaks of Cr _2*p*_ at 578.68 eV, evincing the occurrence of the uptake process (Fig. 8A). Zeta potential elucidated the abundance of positive active species (26.6 mV) on the ATP–CPBr@CA surface at pH 2. Consequently, the cationic adsorption sites of the beads could capture the anionic Cr (VI) by the potent electrostatic interactions. The peaks shift of the N_1s_-spectrum is most likely due to the contribution of the protonated N^+^ of CPBr in the electrostatic interaction (Fig. 8B). Notably, the electrons transferred from the distributed OH onto the surface of ATP–CPBr@CA could reduce the detrimental Cr (VI) to the less toxic Cr (III). Then, the produced Cr (III) ions are attached to the beads via coordination-covalent bonds. These suggestions were proved from the Cr_2p_ spectrum (Fig. 8C) that showed the distinguished peaks of Cr (VI) at 589.13 and 580.54 eV and Cr (III) at 585.6 and 577.39 eV. Noteworthy, the amount of adsorbed Cr (VI) and Cr (III) onto the ATP–CPBr@CA was 31.32% and 54.75%, respectively, indicating the significant role of the reduction reaction in the removal of Cr (VI).

Moreover, the partial ion exchange could participate in the adsorption of Cr (VI) in the solution in which the anionic Br_3*p*_ could partially replace by Cr (VI) as well as Cr (III) partially exchange with Al_2*p*_, Si_2*p*_, and Mg_1*s*_ cations. The XPS survey confirmed the probability of the ion exchange mechanism since there was a noticeable decline in the ratios of Br_3*p*_, Al_2*p*_, Si_2*p*_, and Mg_1*s*_. Furthermore, the possibility of forming outer-sphere complexation between the Cr (VI) and OH groups onto the surface of beads. In addition, the oxygen-containing attapulgite could form inner-sphere complexation with Cr (VI). The peak shifting in the O_1*s*_-spectrum (Fig. 8D) asserted the participation of the oxygenated functional groups of ATP–CPBr@CA in the adsorption of Cr (VI) by outer- and inner-sphere complexations. Interestingly, the interconnect pores structure of ATP–CPBr@CA beads which is the unique feature of the CA beads, provides a pore-filling mechanism during the adsorption process, where the Cr (VI) ions could penetrate the pores.

**Figure 9:**
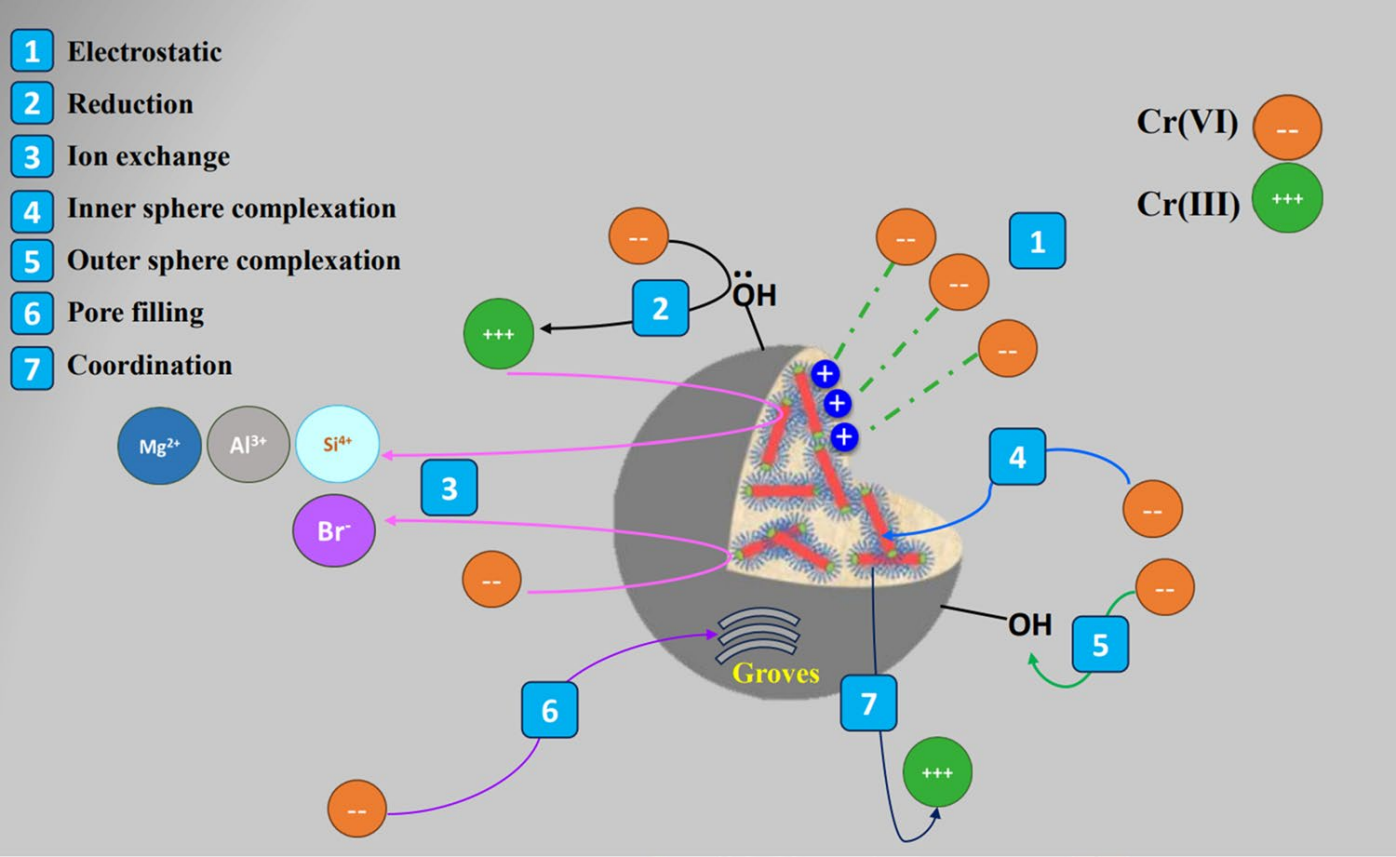
Removal mechanism of Cr (VI) ions by ATP–CPBr@CA composite beads.

In one word, the primer adsorption capacity of ATP–CPBr@CA beads toward Cr (VI) that attained 302.11 mg/g is most probably due to the participation of varied powerful physical and chemical interactions in the Cr (VI)/ATP–CPBr@CA adsorption system, comprising electrostatic interaction, reduction reaction, ion exchange, outer-sphere complexation, pore-filling, and inner-sphere complexation (Fig. 9).

#### Comparison the adsorption performance with other adsorbents

To evaluate the synthesized ATP–CPBr@CA composite beads’ has ability to absorb Cr (VI) in comparison to other known adsorbents. The better adsorption behaviour of the produced ATP–CPBr@CA composite beads was shown in Table 4. The developed ATP–CPBr@CA composite beads thus provide extremely effective adsorbent material for heavy metal ions decontamination from the polluted wastewater.

**Table 4:** Comparable investigation for Cr (VI) onto ATP–CPBr@CA composite beads and other reported adsorbents.

Adsorbent	q_max_ (mg/g)	Eq. time (min)	Refs.
MAC–ATP composite	119.62	120	^39^
FCA/SiO_2_ membrane	19.45	40	^40^
Fe_3_O_4_/ZIF-67@AmCs beads	119.05	80	^41^
ATP–supported nZVI composite	266.65	720	^42^
TEPA-Alg beads	77.00	180	^43^
Fe_3_O_4_-coated CA/CS nanofibers	193.20	300	^46^
CA–PCL/CS nanofiber	126.00	150	^49^
ATP–CPBr@CA composite beads	302	120	This study

#### Reusability of spent ATP–CPBr@CA

**Figure 10:**
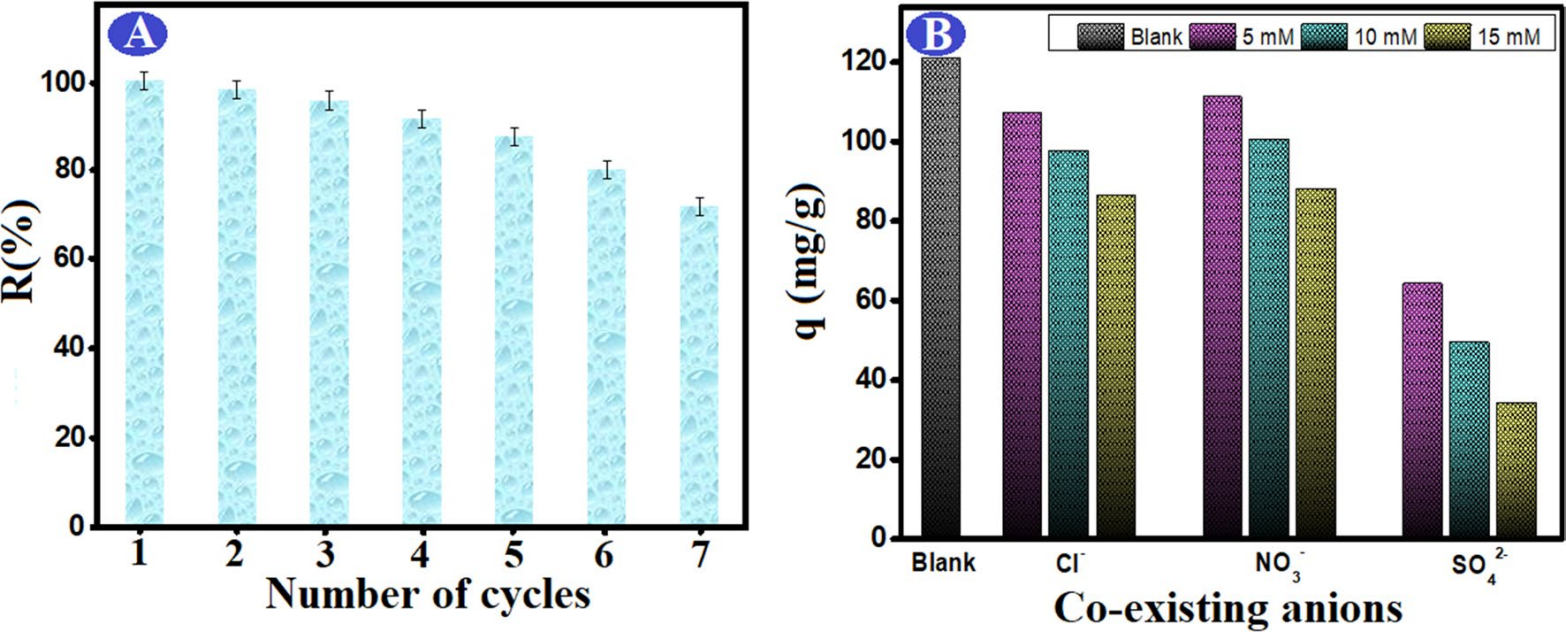
(**A**) Regeneration of ATP–CPBr@CA composite beads in the adsorption of Cr (VI) and (**B**) the influence of co-existing anions on the Cr (V) adsorption capacity.

From economic point of view, it is essential to examine the reusability of the constructed adsorbent^58^. Figure 10A elucidated that the ATP–CPBr@CA composite beads still retain better adsorption properties after seven succeeding cycles. It was observed that the composite beads only lost about 4.37% from their initial efficiency, while the overall efficiency exceeded 70% after the seventh cycle. These findings prove that the composite beads have high stability in water with acceptable removal reactivity for several adsorption–desorption cycles, suggesting their potential applicability as a reusable adsorbent for Cr (VI) ions with high performance.

#### Effect of co-existing anions

The effects of co-existing Cl^−^, NO_3_^−^and SO_4_^2−^ anions using same concentrations of 5, 10, and 15 mM on the adsorption capability of Cr (VI) ions by the as fabricated ATP–CPBr@CA were examined. Generally, Fig. 10B showed that when the concentration of coexisting anions increased, the rejection efficiency decreased. This may be due to the formation of the complex generated by the interaction of co-existing anions with the ions from ATP–CPBr@CA, which reduces the surface reactive sites of ATP–CPBr@CA, and as a result, reducing the removal percentage of Cr (VI) ions^59^. As demonstrated, each of the studied three anions, all obstructs the adsorption of Cr (VI) to a varying extent. In the ATP–CPBr@CA system, the suppression of reactivity to Cr (VI) adsorption occurs in the following order: SO_4_^2−^  > NO_3_^−^  > Cl^−^. Additionally, the SO_4_^2−^ anions displayed the most suppression consequence, due to the strong aptitude of SO_4_^2−^ anions for ion competition with Cr_2_O_7_^2−^, resulting in a decrease in the available adsorption sites and subsequently decrease the removal rate of Cr (VI) ions^60,61^. Moreover, the removal of Cr (VI) ions by ATP–CPBr@CA composite beads was inhibited by Cl^−^ and NO_3_^−^ anions.

## Conclusion

This study reported the construction and adsorbability of a new ATP–CPBr@CA composite for the adsorptive removal of Cr (VI) ions. The developed composite was formulated in in the form of easy-separable beads via a low-cost and simple technique. The successful formulation of the composite beads was evidenced by several analysis tools. Parameters affecting the adsorption process were explored thorough a series of batch adsorption studies Likewise, several kinetics and isotherms studies were performed to explicate the adsorption process. The removal efficiency of pure CA beads was greatly augmented from 21.46 to 71.84% after incorporation of ATP–CPBr. According to Langmuir model, maximum monolayer adsorption capacity of 302 mg/g was accomplished at pH 2, while Temkin model denoted that the adsorption process of Cr (VI) ions onto ATP–CPBr@CA composite beads was categorized by a uniform distribution of the binding energies. Kinetically, the gained data obeyed the pseudo 2nd order kinetic model, while the intraparticle diffusion model verified two stages for diffusion. The removal of Cr (VI) ions primarily involves adsorption, reduction, and co-precipitation. Besides, the reusability results attested the potential proficiency of ATP–CPBr@CA composite beads to adsorb Cr (VI) ions for seven repeated cycles with higher performance. In conclusion, the higher adsorption performance, simple processing, ease-separation, and better renewability strongly recommend the potential usage of the formulated ATP–CPBr@CA composite beads as sustainable candidate for removing anionic Cr (VI) ions from contaminated water.

### Supplementary Information

Supplementary Information.

## Data Availability

The datasets used and/or analysed during the current study available from the corresponding author on reasonable request.
