# Supplementary material for: Construction of attapulgite decorated cetylpyridinium bromide/cellulose acetate composite beads for removal of Cr (VI) ions with emphasis on mechanistic insights
Source: Sci Rep. 2024 May 28;14:12164. doi: 10.1038/s41598-024-62378-4 (PMC11133475; doi:10.1038/s41598-024-62378-4)
Supplement: Supplementary file 1 — Supplementary Information. [file 41598_2024_62378_MOESM1_ESM.docx]

**Supplementary information**

**S.1. Instrumental characterization**

Fourier Transform Infrared spectra (FTIR; Tensor II, Bruker, USA) and Scanning Electron Microscope (SEM; Joel, Jsm-IT200, Japan) were employed to investigate the chemical structure and morphological properties of the composite beads, respectively. Zeta potential (ZP; Malvern, UK) was used to examine the surface charges. Moreover, the elemental composition and the crystallite phase of ATP-CPC@CA composite beads was scrutinized by the X-ray photoelectron spectroscopy (XPS; Thermo-scientific, 250Xi VG, USA) and X-ray diffractometer (XRD; Bruker D8 ADVANCE, Germany), respectively.


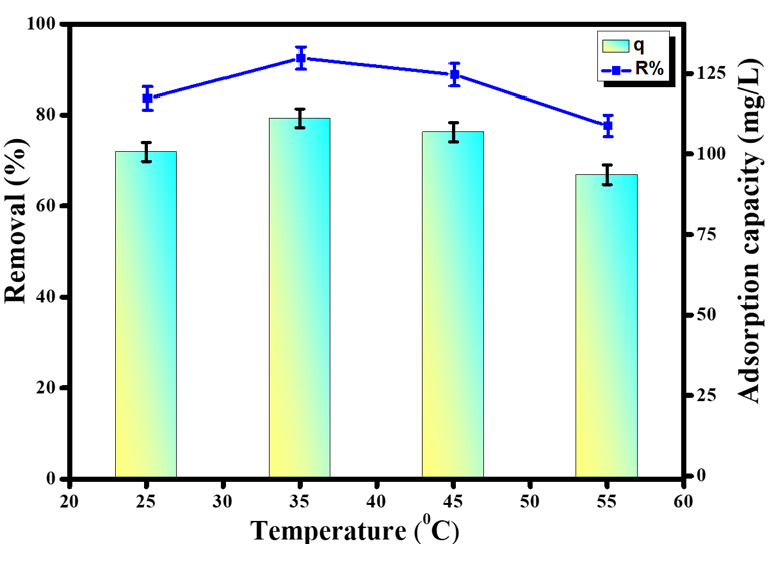


**Fig. S1**. Impact of system temperature on the removal (%) and the adsorption capacity of Cr (VI) onto ATP-CPC@CA composite beads at constant [dose 0.015 g, contact time 120 min, pH 2, and initial Cr (VI) concentration 100 mg/L].
